# Experimental Malaria in Pregnancy Induces Neurocognitive Injury in Uninfected Offspring via a C5a-C5a Receptor Dependent Pathway

**DOI:** 10.1371/journal.ppat.1005140

**Published:** 2015-09-24

**Authors:** Chloë R. McDonald, Lindsay S. Cahill, Keith T. Ho, Jimmy Yang, Hani Kim, Karlee L. Silver, Peter A. Ward, Howard T. Mount, W. Conrad Liles, John G. Sled, Kevin C. Kain

**Affiliations:** 1 Institute of Medical Science, University of Toronto, Toronto, Ontario, Canada; 2 SAR Laboratories, Sandra Rotman Centre for Global Health, University Health Network-Toronto General Hospital, University of Toronto, Toronto, Ontario, Canada; 3 Mouse Imaging Centre, Hospital for Sick Children, Toronto, Ontario, Canada; 4 Department of Medical Biophysics, University of Toronto, Toronto, Ontario, Canada; 5 Division of Neurology, Department of Psychiatry, University of Toronto, Toronto, Ontario, Canada; 6 Department of Pathology, University of Michigan, Ann Arbor, Michigan, United States of America; 7 Department of Medicine, University of Washington, Seattle, Washington, United States of America; 8 Tropical Disease Unit, Division of Infectious Diseases, Department of Medicine, University of Toronto, Toronto, Ontario, Canada; Faculdade de Medicina da Universidade de Lisboa, PORTUGAL

## Abstract

The *in utero* environment profoundly impacts childhood neurodevelopment and behaviour. A substantial proportion of pregnancies in Africa are at risk of malaria in pregnancy (MIP) however the impact of *in utero* exposure to MIP on fetal neurodevelopment is unknown. Complement activation, in particular C5a, may contribute to neuropathology and adverse outcomes during MIP. We used an experimental model of MIP and standardized neurocognitive testing, MRI, micro-CT and HPLC analysis of neurotransmitter levels, to test the hypothesis that *in utero* exposure to malaria alters neurodevelopment through a C5a-C5aR dependent pathway. We show that malaria-exposed offspring have persistent neurocognitive deficits in memory and affective-like behaviour compared to unexposed controls. These deficits were associated with reduced regional brain levels of major biogenic amines and BDNF that were rescued by disruption of C5a-C5aR signaling using genetic and functional approaches. Our results demonstrate that experimental MIP induces neurocognitive deficits in offspring and suggest novel targets for intervention.

## Author Summary

A growing body of evidence has established the importance of the *in utero* environment on neurodevelopment and long-term cognitive and behavioral outcomes. These data suggest factors that disrupt the tightly regulated *in utero* environment can modify normal neurodevelopmental processes. Approximately 125 million pregnancies worldwide are at risk of malaria infection every year. However the impact of *in utero* exposure to MIP on fetal neurodevelopment is unknown. Here we use a mouse model of malaria in pregnancy to examine the impact of maternal malaria exposure on neurocognitive outcomes in offspring. We observed impaired learning and memory and depressive-like behavior in malaria-exposed offspring that were neither congenitally infected nor low birth weight. These neurocognitive impairments were associated with decreased tissue levels of neurotransmitters in regions of the brain linked to the observed deficits. Disruption of maternal C5a complement receptor signaling restored the levels of neurotransmitters and rescued the associated cognitive phenotype observed in malaria-exposed offspring. This study provides the first evidence implicating a causal link between pre-natal exposure to malaria, complement signaling and subsequent neurocognitive impairment in offspring.

## Introduction

Each year, an estimated 125 million pregnancies worldwide are at risk of malaria infection [1]. *Plasmodium falciparum* infections during pregnancy are more frequent, and associated with higher parasite burdens and worse clinical outcomes than those of non-pregnant individuals [2,3]. MIP has profound maternal and fetal health consequences including increased risk of maternal anemia, preterm birth, stillbirth, fetal growth restriction (FGR) and low birth weight infants (LBW), resulting in an estimated 200,000 infant deaths annually [4]. MIP is characterized by the accumulation of parasitized erythrocytes (PEs) and monocytes/macrophages in the placenta [2,3]. While it is believed that this localized placental immune response contributes to adverse birth outcomes, the precise mechanism by which parasite and monocyte accumulation in the placenta results in poor pregnancy outcomes remains unknown. Recent evidence supports a role for altered angiogenesis and resulting placental vascular insufficiency [5,6].

The complement system is an essential component of the innate immune response to microbial pathogens [7–9]. Excessive complement activation, notably generation of the anaphylatoxin C5a, has been implicated in mediating deleterious host responses and poor clinical outcomes to infections [8,10]. Malaria infection is known to induce activation of the complement system through multiple pathways, and recent studies support a mechanistic role for C5a in the pathophysiology of severe malaria and malaria in pregnancy [10–14]. Complement activation has also been proposed as a common pathway mediating adverse pregnancy outcomes in the absence of infection [15,16]. Excessive C5a generation was implicated as a mediator of placental injury in murine models of spontaneous miscarriage and FGR [17]. Moreover, human studies have associated complement split products (e.g. C3a, C5a) with pregnancy complications [18,19].

Recent evidence has also identified an essential role for the complement system in both normal and abnormal neurodevelopmental processes [20–22]. Complement proteins and their receptors are widely expressed within the central nervous system and play a major role in regulating normal synaptic development and function [23].

Alterations in the *in utero* environment as a result of maternal infection may have profound and long-term implications for the developing fetus. Recent studies indicate that immunological stress at the maternal-fetal interface can alter later-life brain development and behaviour [24,25]. Despite the potential public health implications, little is known about the impact of *in utero* exposure to MIP on fetal and infant neurological development. Based on the above evidence implicating C5a in both neurodevelopment and MIP-associated adverse birth outcomes, we tested the hypothesis that *in utero* exposure to experimental MIP (EMIP) alters offspring neurodevelopment and that disruption of maternal C5a receptor (C5aR) signaling would rescue EMIP-induced neurocognitive injury in exposed offspring.

## Results

### Lower parasite inoculum eliminates low birth weight (LBW) as a confounder in experimental malaria in pregnancy (EMIP)

**Fig 1:**
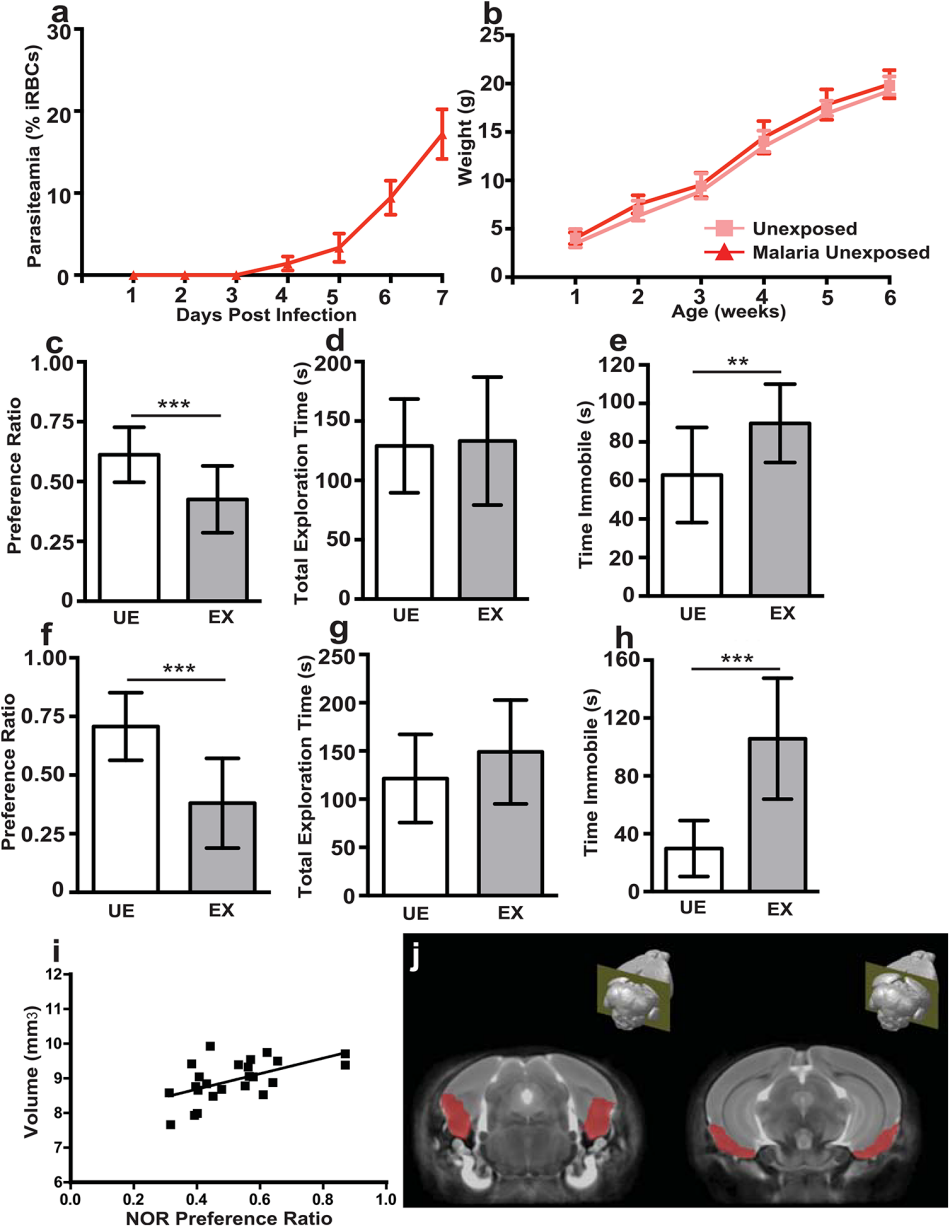
*In utero* exposure to EMIP induces a persistent neurocognitive phenotype in offspring but is not associated with regional volumetric anatomical changes determined by MRI. **(a)** Maternal parasitaemia (day one to seven post infection, n = 5) expressed as percent of infected red blood cells (iRBCs) per total red blood cells counted. **(b)** Offspring weight from one to six weeks of age in unexposed (n = 15) and malaria exposed offspring (n = 13). **(c)** Testing performance (preference ratio) and **(d)** total exploration time of unexposed (UE, n = 15) and malaria exposed (EX, n = 13) offspring (6 weeks of age) in the NOR test. **(e)** Performance of UE (n = 15) and EX (n = 15) offspring (6 weeks of age) in the TST test. **(f)** Testing performance (preference ratio) and **(g)** total exploration time of unexposed (UE, n = 11) and malaria exposed (EX, n = 12) offspring tested at 20 weeks of age in the NOR test. **(h)** Performance of UE (n = 11) and EX (n = 12) offspring in the TST test at 20 weeks of age. **(i)** Correlation between entorhinal cortical volume and performance (preference ratio) in the NOR test of unexposed and malaria exposed offspring (n = 24, spearman rho = 0.4912, *P* = 0.0044). **(j)** Depiction of average MRI, generated from all scans and all experimental groups, and outline of area used to define the entorhinal cortex. **P < 0.01, ***P < 0.005; T-Test. Data are presented as mean +/- SD.

LBW, as a result of preterm birth or FGR, is known to be associated with impaired neurocognitive development [26,27]. Since MIP may cause LBW, these infants would be expected to experience an increased risk of neurocognitive impairment; however the majority of fetuses exposed to malaria *in utero* do not develop LBW. Therefore, in order to avoid LBW as a confounder and isolate the effects of malaria exposure alone on offspring neurodevelopment, we reduced the inoculum given to dams in a validated model of EMIP [28] from 10^6^ to 10^5^ PEs. This inoculum was associated with the presence of parasitized erythrocytes in the placenta and localized inflammation in the placenta (S1 Fig and S2 Fig). However the 10^5^ inoculum was associated with lower maternal peripheral parasitemia (Fig 1A) and less marked placental pathology than that previously reported with a dose of 10^6^ PEs [28]. This modification eliminated the LBW phenotype in this model and resulted in equivalent birth weights (from 1 to 20 weeks of age) in control pups compared to offspring exposed *in utero* to EMIP (Figs 1b, 5b and S3 Fig, S4 Fig and S5 Fig). No significant differences were observed in the length of gestation or litter size in this lower inoculum EMIP model (S1 Table). Placentas from malaria-infected litters (wild-type and *C5ar*-/-) showed placental inflammation as indicated by increased expression of tumor necrosis factor (TNF), interferon gamma (IFN_ϒ_), intracellular adhesion molecule-1 (ICAM-1) and monocyte chemotactic protein 1 (MCP-1, CCL2) (S2 Fig, p < 0.05). Wild-type mice showed increased expression of ICAM and reduced expression of MCP in comparison with *C5ar-/-* mice in placentas from both uninfected and malaria-infected litters (S2 Fig, p < 0.05). Absence of congenital infection was confirmed by blood smears and PCR of fetal blood.

### *In utero* exposure to EMIP is associated with persistent neurocognitive deficits in offspring

To investigate the impact of *in utero* EMIP-exposure on neurocognitive performance, we compared EMIP-exposed pups to unexposed controls using a battery of standardized neurocognitive tests [29–31]. Exposed offspring showed impaired novel object recognition (NOR) in the NOR test of non-spatial learning and memory, and increased immobility in the tail suspension test (TST), a test of depressive-like behavior. Performance in the NOR test was impaired in EMIP-exposed offspring compared with unexposed offspring (*P* = 0.0004; Fig 1C). Differences observed between groups could not be attributed to other behavioral factors including differences in time of initial exploration of objects or motor behavior during testing (Fig 1D, S3 Fig). Immobility in the TST was increased in EMIP-exposed offspring compared with unexposed offspring (*P* = 0.004; Fig 1E). The behavioral deficits persisted to adulthood in EMIP-exposed offspring. Exposed mice tested at 20 weeks of age showed impaired performance in the NOR test (*P* = 0.001; Fig 1F) and increased immobility in the TST (*P* = 0.0002; Fig 1H).

### *In utero* EMIP-exposure is not associated with changes in regional brain volumes by MRI

We performed MRI to determine if the observed neurocognitive phenotype in EMIP-exposed mice was associated with changes in regional brain volumes. Prior to imaging, all mice were tested in the NOR test to confirm their behavioral phenotype. *In utero* exposed offspring showed impaired performance in the NOR test compared with unexposed offspring (*P* = 0.0009; S3 Fig). Volumetric analysis of brain volume in 63 distinct regions revealed no differences between EMIP-exposed and control mice (S2 Table).

A significant correlation across all WT mice (exposed and unexposed) was observed between total entorhinal cortical volume (volume of left and right cortices together) and performance in the NOR test (Spearman’s rho, 0.4912, *P* = 0.0044; Fig 1I). The mouse brain atlas [32] used to define the entorhinal cortex is depicted in Fig 1J. These data confirm a role for the entorhinal cortex in performance in the NOR test as suggested by previous reports [33,34]; however no difference was observed between malaria-exposed and unexposed animals.

### EMIP-exposure is associated with altered fetal neurovascular development as determined by micro-CT imaging

**Fig 2:**
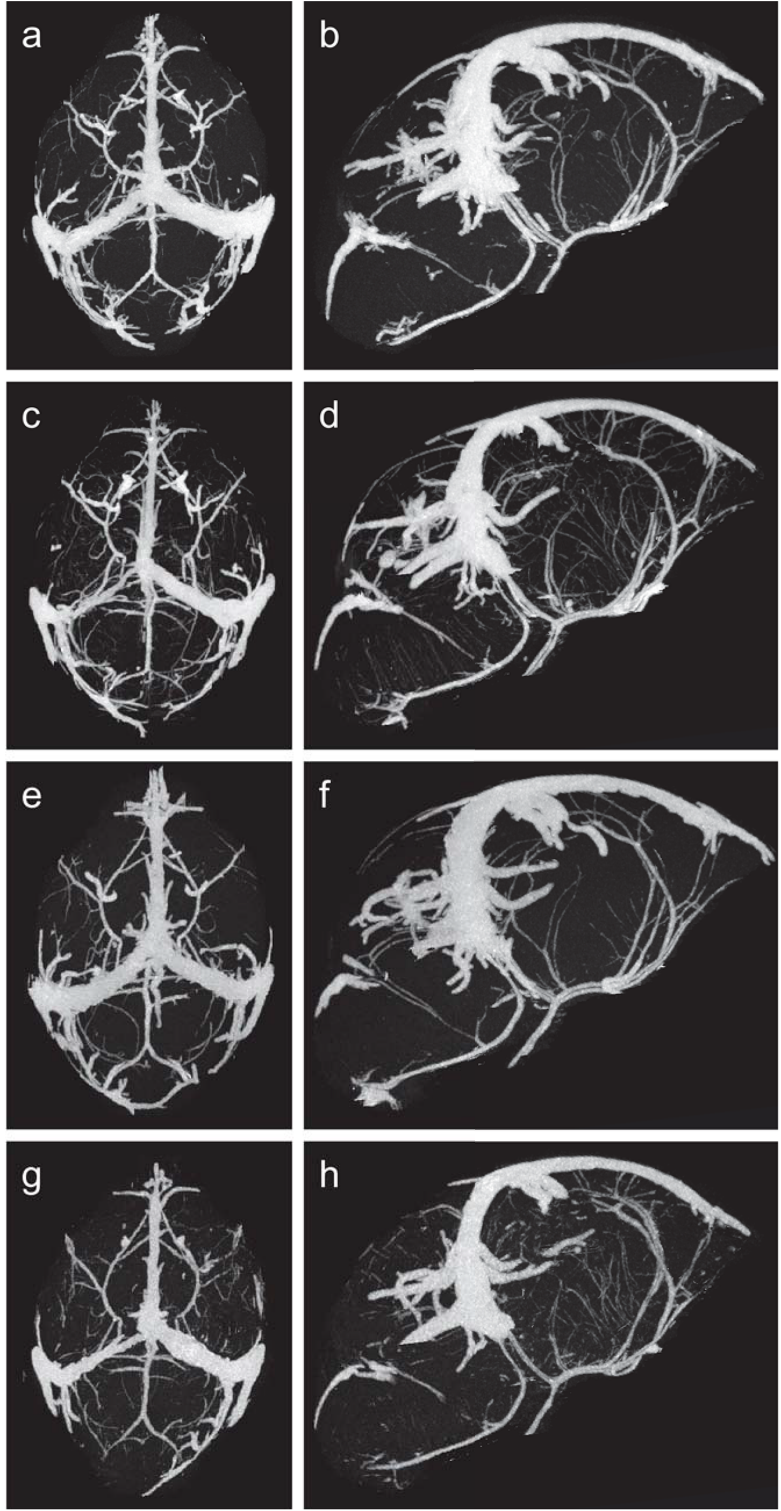
Micro-CT images of fetal cerebral vasculature at gestational day 18. Maximum intensity projection renderings of the axial **(a)** and sagittal **(b)** view of vasculature from wild-type unexposed offspring, axial **(c)** and sagittal **(d)** view of vasculature from wild-type malaria exposed offspring, axial **(e)** and sagittal **(f)** view of vasculature from *C5ar-/-* unexposed offspring and axial **(g)** and sagittal **(h)** view of vasculature from *C5ar-/-* malaria exposed offspring.

**Fig 3:**
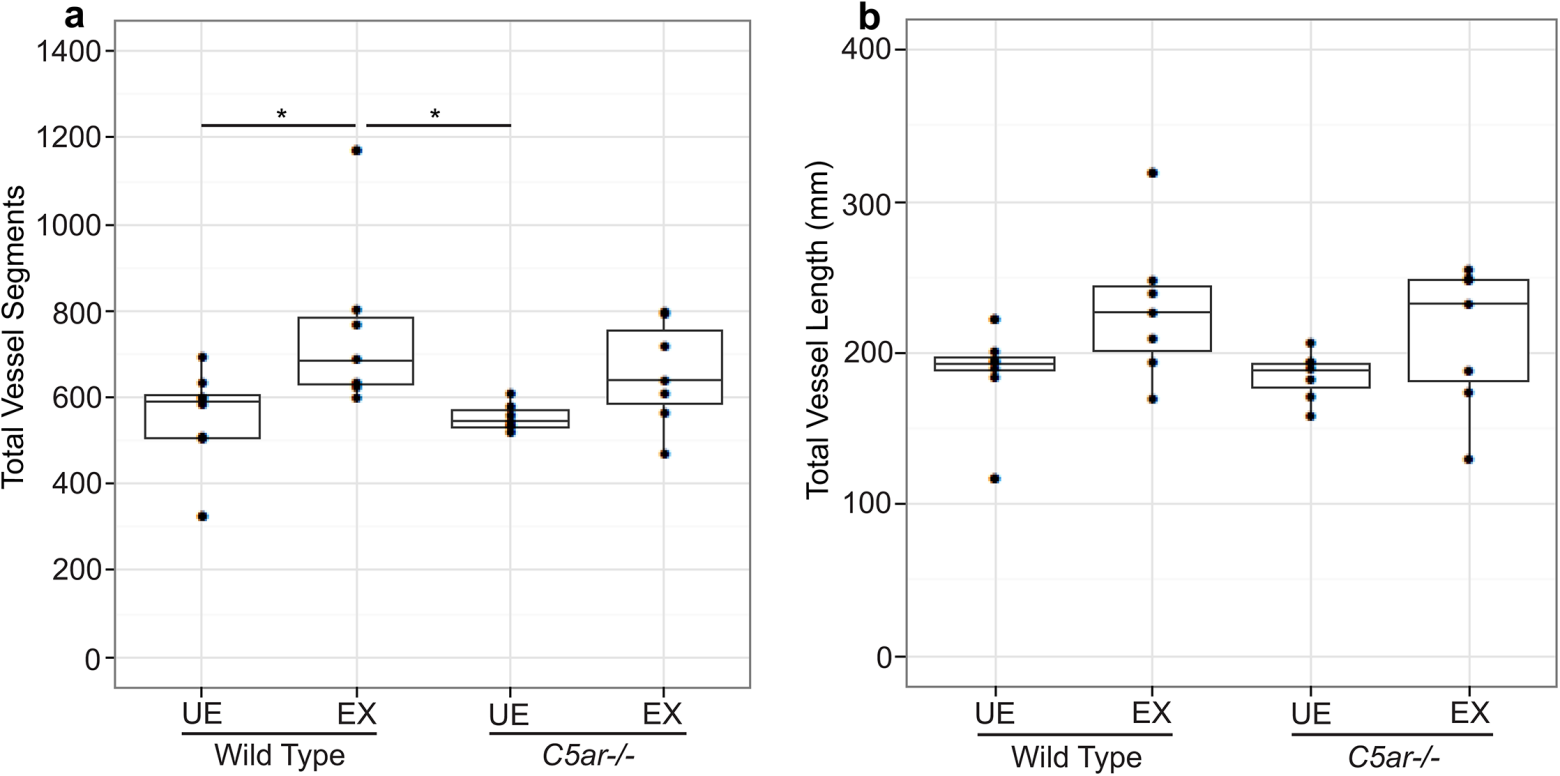
Quantitative analysis of fetal cerebral vasculature using a vessel segmentation algorithm determined (a) the number of vessel segments and (b) the total length of all vessel segments (mm) of wild-type unexposed (WT UE, n = 8), wild-type malaria exposed (WT EX, n = 7), C5a receptor knockout unexposed (*C5ar-/-* UE, n = 7) and C5a receptor knockout malaria-exposed (*C5ar-/-* EX, n = 7) offspring. *P < 0.*05*; ANCOVA and post-test. Box plots depict median and interquartile range.

Previous studies have shown that malaria in pregnancy is associated with altered placental vascular development [5]. We hypothesized that fetal cerebral vasculature may also be modified in malaria-exposed offspring, and that altered cerebrovascular development may contribute to the observed neurocognitive phenotype. Using a novel imaging approach in fetal mice, we performed micro-CT scans of fetal cerebral vasculature at G18. To our knowledge, this is the first time micro-CT has been used to visualize fetal cerebral vasculature. Using this technique, we identified all major cerebral vessels in fetuses and determined that there were no qualitative differences in major vessel architecture (Fig 2A–2D, S3 Table). In order to assess the impact of malaria–exposure on small vessel development, we further examined fetal cerebral vasculature with automated vessel tracking of the 3D images [35]. Vessel tracking analysis revealed a significant increase in the total number of vessel segments associated with *in utero* malaria-exposure (Fig 3A, *P* < 0.05). Malaria-exposure did not result in significant changes to total vessel length (Fig 3B).

### EMIP-exposure is associated with regional decreases in brain tissue levels of major biogenic amines

**Fig 4:**
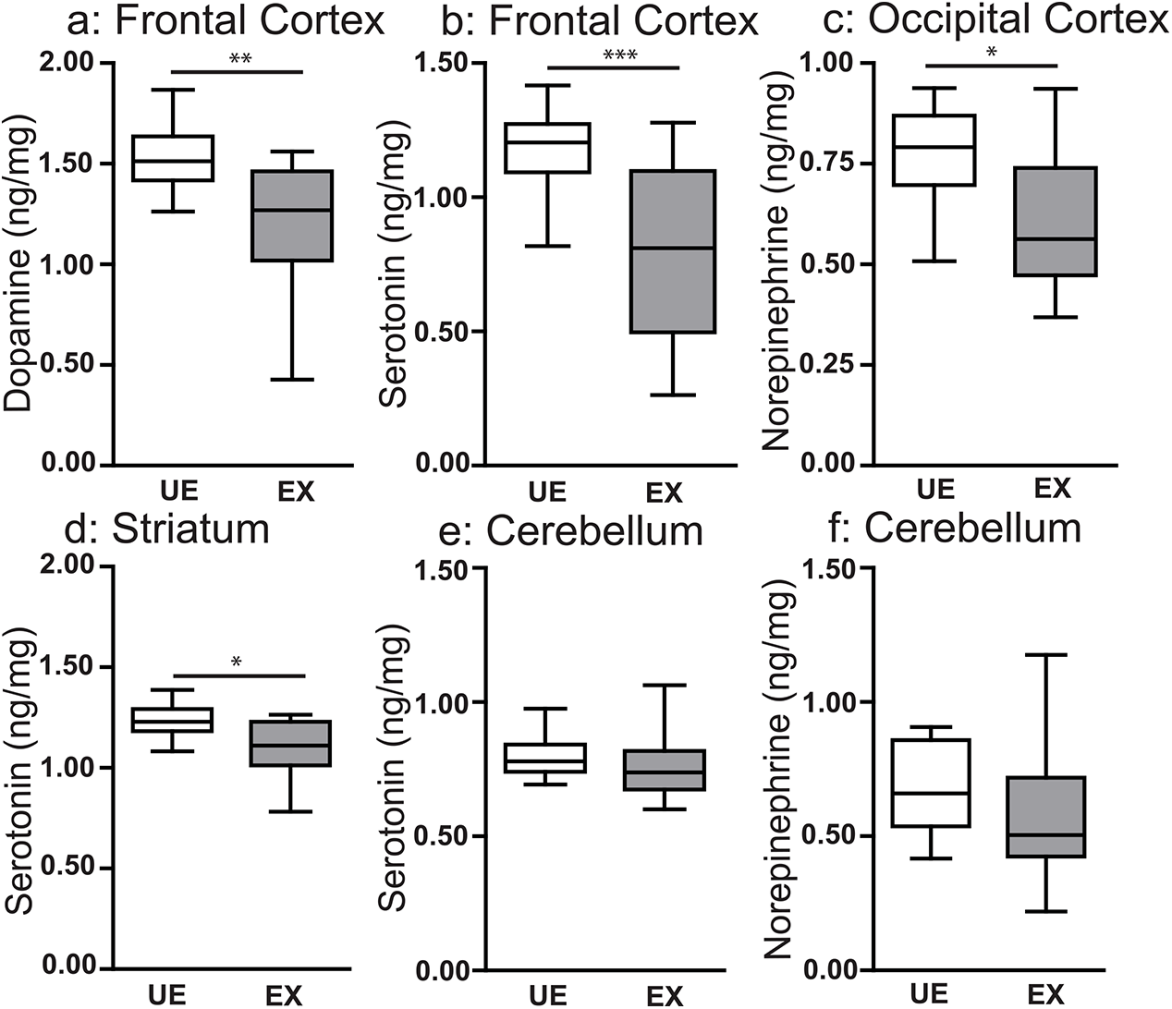
*In utero* exposure to EMIP is associated with localized changes in tissue levels of major biogenic amines in offspring at 8 weeks of age. Tissue levels of **(a)** dopamine, and **(b)** serotonin in the frontal cortex, **(c)** norepinephrine in the temporoparietal cortex, **(d)** serotonin in the striatum, **(e)** serotonin and **(f)** norepinephrine in the cerebellum of unexposed (UE, n = 15) and malaria exposed (EX, n = 15) offspring. * *P < 0*.*05*, **P < 0.01, ***P < 0.005; T-Test. Box plots depict median, 95% confidence interval (box) and range (whiskers).

Examination by MRI or micro-CT may not be sufficiently sensitive to detect subtle neurological features, such as changes in neuronal connectivity, capable of altering neurocognitive outcomes. Therefore, we next investigated levels of biogenic amine transmitters (dopamine, norepinephrine and serotonin) in four regions of interest (frontal cortex, temporoparietal cortex, striatum and hippocampus) based on their previously established involvement in the behavioral phenotypes we observed [36–38]. All tissue was harvested from animals that had been tested behaviorally to confirm their phenotype (Fig 1C–1E). Wild-type malaria-exposed offspring showed decreased tissue levels of dopamine (*P* < 0.01; Fig 4A) and serotonin (*P* < 0.005; Fig 4B) in the frontal cortex, norephinephrine in the temporoparietal cortex (*P* < 0.05; Fig 4C) and serotonin in the striatum (*P* < 0.05; Fig 4D) compared with wild-type unexposed offspring. Tissue levels of the catecholamine metabolite homovanillic acid were reduced in the frontal cortex and hippocampus of wild-type exposed mice (*P* < 0.05; S4 Table). Tissue levels of these analytes in each of the regions tested are reported in S4 Table. Maternal peripheral parasitemia (ranging from 14–31% on the day of delivery; Fig 1A) was not associated with differences in the observed levels of major biogenic amines, MRI or micro-CT imaging or neurocognitive outcomes.

### Genetic disruption of C5a-C5aR signaling in dams rescues neurocognitive deficits in EMIP-exposed offspring

**Fig 5:**
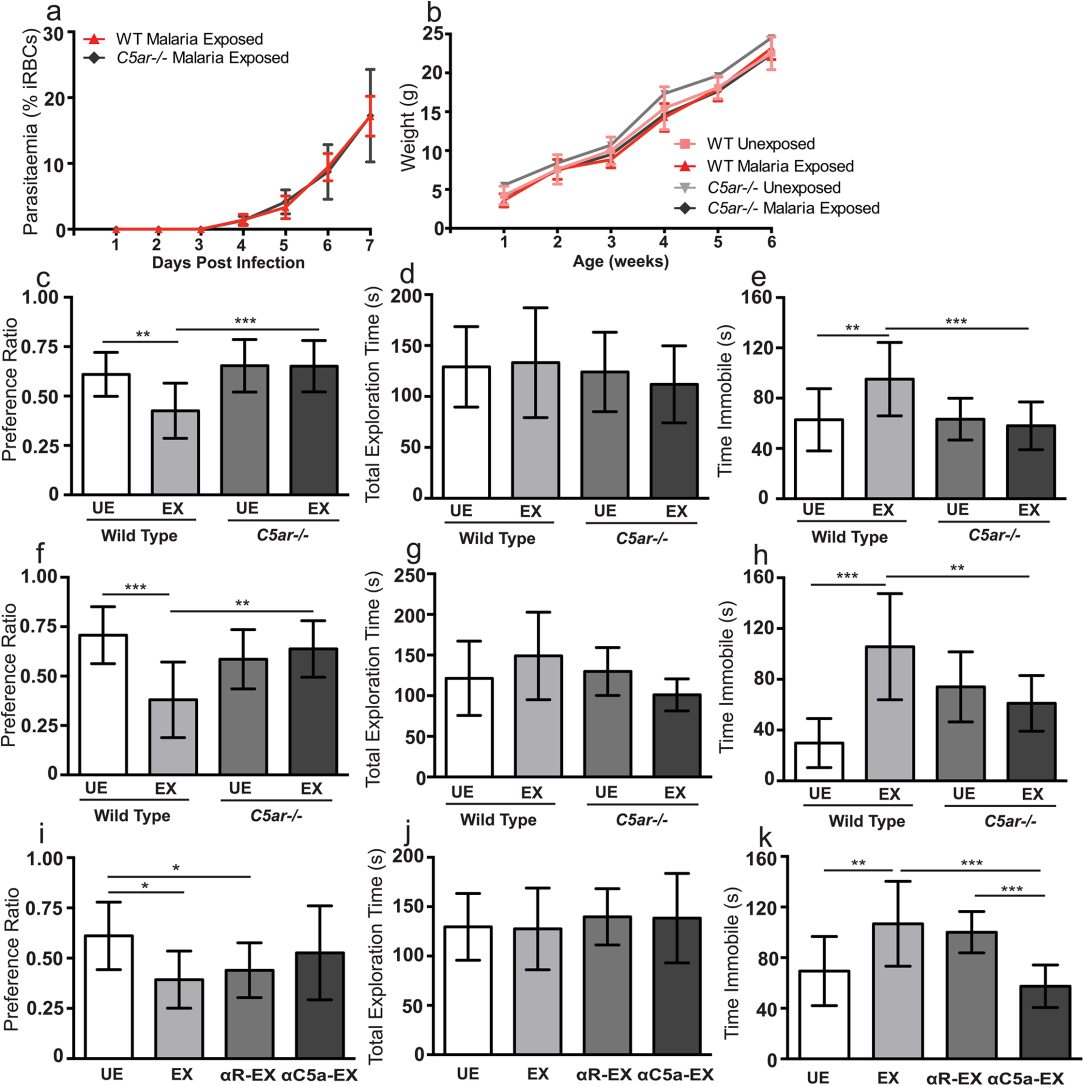
*In utero* exposure to EMIP induces a cognitive phenotype in offspring that is rescued by genetic and pharmacological blockade of C5a-C5a receptor signaling. **(a)** Maternal parasitaemia (day one to seven post infection) expressed as percent of infected red blood cells (iRBCs) per total red blood cells counted in wild-type (n = 5) and *C5ar-/-* dams (n = 5). **(b)** Weight from one to six weeks of age in wild-type malaria unexposed (n = 14) and wild-type malaria exposed offspring (n = 13), *C5ar-/-* malaria unexposed (n = 13) and *C5ar-/-* malaria exposed (n = 14) offspring. **(c)** Testing Performance (preference ratio) and **(d)** total exploration time of wild-type unexposed (WT UE, n = 14), wild-type malaria exposed (WT EX, n = 13), C5a receptor knockout unexposed (*C5ar-/-* UE, n = 13) and *C5ar-/-* malaria exposed (*C5ar-/-* EX, n = 14) offspring in the NOR test (*P* > 0.05). **(e)** Performance of WT UE (n = 15), WT EX (n = 15), *C5ar-/-* UE (n = 13) and *C5ar-/-* EX (n = 15) offspring in the TST test. (**f)** Testing Performance (preference ratio) and **(g)** total exploration time of wild-type unexposed (WT UE, n = 11), wild-type malaria exposed (WT EX, n = 12), C5a receptor knockout unexposed (*C5ar-/-* UE, n = 13) and *C5ar-/-* malaria exposed (*C5ar-/-* EX, n = 9) offspring tested at 20 weeks of age in the NOR test. **(h)** Performance of WT UE (n = 12), WT EX (n = 11), *C5ar-/-* UE (n = 12) and *C5ar-/-* EX (n = 10) offspring tested at 20 weeks of age in the TST test. **(i)** Testing Performance (preference ratio) and **(j)** total exploration time of UE offspring (n = 11), EX offspring (n = 11), EX offspring of control rabbit antisera treated dams (R-EX, n = 12) and EX offspring of C5a antisera treated dams (αC5a-EX, n = 12) offspring in the NOR test (*P* > 0.05 for total exploration time). **(k)** Performance of UE (n = 11), EX (n = 12), R-EX (n = 10) and αC5a EX (n = 12) offspring in the TST test. *P < 0.05, **P < 0.01, ***P < 0.005; one-way ANOVA and post-test. Data are presented as mean +/- SD.

Based on evidence linking C5a to both neuropathology and the pathophysiology of malaria [5,11,13,14], we examined the impact of genetic disruption of the C5a-C5aR signaling on neurocognitive outcomes in EMIP-exposed offspring. The deficits in NOR performance observed in WT EMIP-exposed offspring were completely rescued in C5aR deficient (*C5ar-/-)* EMIP-exposed offspring (*P* < 0.001) (one-way ANOVA and post-test, *P* < 0.004; Fig 5C). Again, no differences in time of initial exploration or motor behaviour were observed (Fig 5D, S4 Fig). Similarly, although immobility was increased in EMIP-exposed WT offspring in the TST, these features of affective-like behaviour were absent in EMIP-exposed offspring where C5aR signaling was disrupted (one-way ANOVA and post-test, *P* < 0.005; Fig 5E). Rescue of the neurocognitive deficits observed in EMIP-exposed *C5ar-/-* offspring persisted to adulthood (Fig 5F–5H). When tested at 20 weeks of age, EMIP-exposed WT mice showed impaired performance in the NOR test compared to EMIP-exposed *C5ar-/-* offspring (*P* < 0.001) and unexposed WT and *C5ar-/-* controls (one-way ANOVA and post-test, *P* < 0.002; Fig 5F). Performance of exposed *C5ar-/-* offspring was similar to unexposed controls (Fig 5F). Adult EMIP-exposed WT offspring, similar to malaria-exposed young mice, showed increased immobility in the tail suspension test compared with unexposed WT offspring (*P* < 0.001), and this effect was rescued in *C5ar-/-* offspring (one-way ANOVA and post-test, *P* < 0.0001; Fig 5H).

### Functional blockade of C5a-C5aR rescues neurocognitive deficits in EMIP-exposed wild-type mice

To provide a separate line of evidence that disruption of C5aR signaling rescues neurocognitive deficits in exposed offspring, we examined the impact of functional blockade of C5a in malaria-infected wild-type dams using C5a antisera [39]. Treatment of dams with anti-C5a antibody rescued the performance of EMIP-exposed offspring in the NOR test and TST. Offspring of dams treated with C5a antisera showed no significant difference in performance compared with unexposed offspring (*P* > 0.05; Fig 5I). However, performance in the NOR test was impaired in EMIP-exposed offspring and exposed offspring of dams treated with control sera (one-way ANOVA, *P* = 0.012; Fig 5I). EMIP-exposed offspring and exposed offspring of dams treated with control sera showed increased immobility in the TST compared with unexposed offspring (*P* < 0.01) and EMIP-exposed offspring of dams treated with C5a antisera (*P* < 0.001) (one-way ANOVA and post-test, *P* < 0.0001; Fig 5K). We performed additional testing on this cohort of animals to examine the impact of the saliency of the stimuli on cognitive performance. No significant difference in freezing behavior (a read out of contextual fear conditioning-based learning) was observed between groups on Day 2 or Day 3 of contextual fear-conditioning (one-way ANOVA and post-test, *P* > 0.05; S5 Fig).

### MRI and micro-CT imaging of fetal cerebral vasculature in EMIP-exposed C5aR deficient mice

C5a has been shown to be directly neurotoxic *in vitro* [22] and blockade of C5aR signaling in experimental models of MIP is associated with increased placental vascular development [5]. Therefore, to begin to examine putative mechanisms by which disruption of C5aR signaling may prevent neurocognitive injury, we performed MRI and micro-CT imaging of fetal cerebral vasculature in unexposed and malaria-exposed *C5ar-/-* offspring. We observed no volumetric changes as determined by MRI (S2 Table) as a result of EMIP-exposure in *C5ar-/-* offspring. Although micro-CT scans of fetal cerebral vasculature (Fig 2E–2H) at G18 revealed a significant increase in total vessel segments in malaria-exposed wild-type offspring (Fig 3A), disruption of C5a-C5aR signaling did not significantly reverse these changes. Therefore, neither changes in brain volumes as determined by MRI, nor microvascular development as assessed by micro-CT, provided an explanation for the cognitive impairments observed and their rescue by C5a-C5aR blockade.

### Disruption of C5aR signaling rescues brain levels of major biogenic amines and brain derived neurotrophic factor (BDNF) in EMIP-exposed offspring

**Fig 6:**
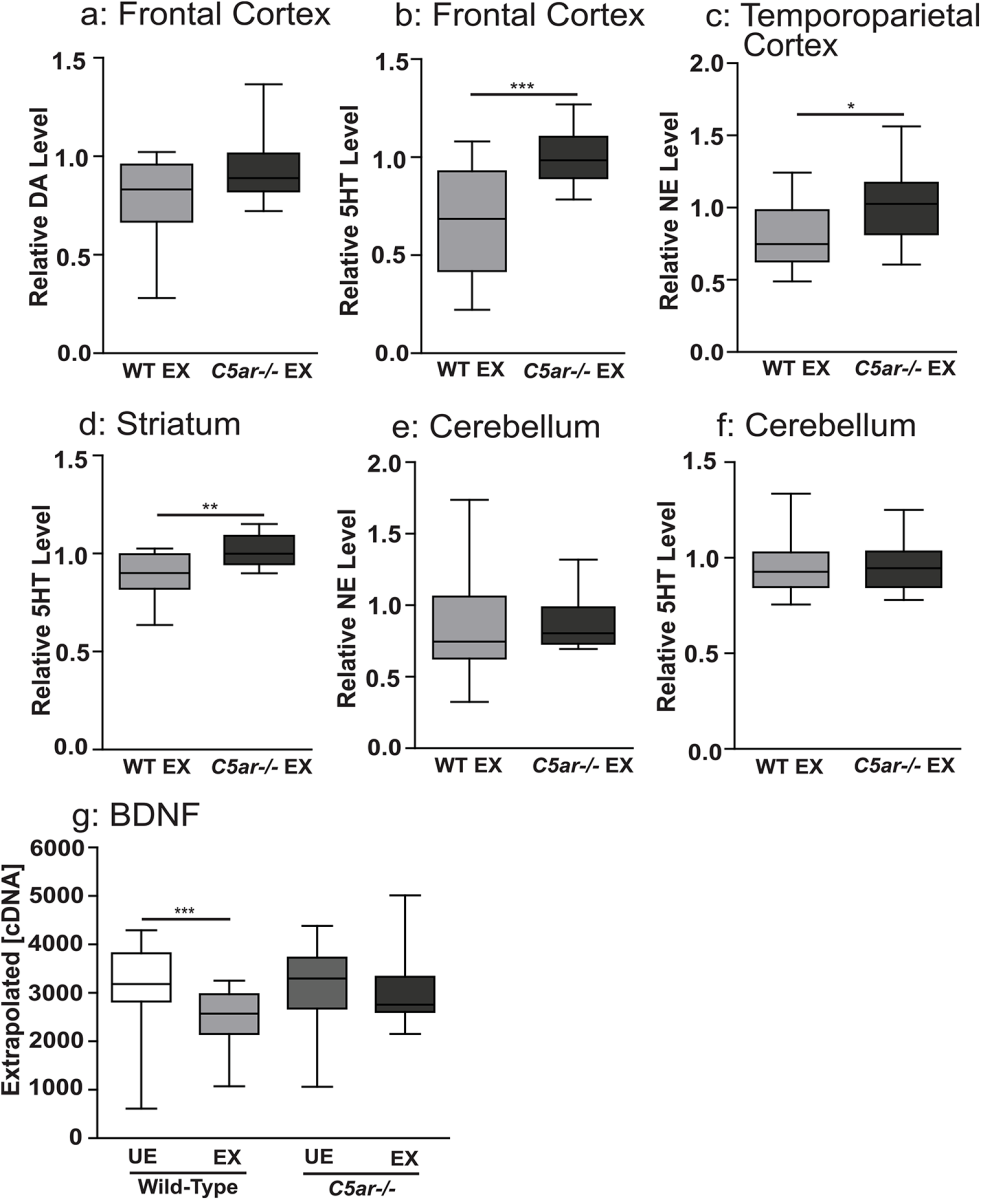
Reduced tissue levels of biogenic amine transmitters were observed in wild-type malaria exposed, but not in C5a receptor knockout offspring, relative to unexposed controls. Brain tissue level of **(a)** dopamine (DA) and **(b)** serotonin (5HT) in the frontal cortex, **(c)** norepinephrine (NE) in the temporoparietal cortex and **(d)** serotonin (5HT) in the striatum, **(e)** norepinephrine and **(f)** serotonin in the cerebellum of wild type malaria exposed offspring (WT EX, n = 15) expressed relative to malaria unexposed wild type offspring and C5a receptor knockout unexposed offspring expressed relative to malaria unexposed *C5ar-/-* offspring (*C5ar-/-*EX, n = 15). *In utero* exposure to EMIP induced dysregulated messenger ribonucleic acid (mRNA) transcription level of BDNF in the fetal brain at gestational day 19. Fetal brain mRNA transcript level expressed as normalized copy number of **(g)** BDNF. *P < 0.05, **P < 0.01, ***P < 0.005; T-test (a-f) and one-way ANOVA (g). Box plots depict median, 95% confidence interval (box) and range (whiskers).

We next extended our analysis to examine the impact of EMIP-exposure on monoamine transmitter levels in adult WT and *C5ar-/-* mice. In contrast to EMIP-exposed WT mice, regional brain levels of biogenic amines were not significantly decreased in EMIP-exposed *C5ar-/-* offspring (*P*>0.05, Students t-test, S5 Table). We normalized the levels of transmitters of EMIP-exposed WT and *C5ar-/-* offspring to the mean of their respective unexposed controls (Fig 6A–6F). Exposed *C5ar-/-* offspring displayed significantly higher levels of serotonin in the frontal cortex (*P* = 0.0028; Fig 6B), norepinephrine in the temporoparietal cortex (*P* = 0.012; Fig 6C) and serotonin in the striatum (*P* = 0.009; Fig 6D), compared to EMIP-exposed WT mice. Given the established role of BDNF in regulating brain monoamine levels [40,41], we determined whether decreased fetal brain BNDF levels were associated with the observed decrease in biogenic amines and whether disruption of C5aR signaling would rescue these levels. We observed decreased BDNF transcript levels in EMIP-exposed WT offspring (Fig 6G); whereas BDNF levels were restored in EMIP-exposed *C5ar-/-* offspring (one-way ANOVA and post-test, *P* <0.001, Fig 6G).

## Discussion

This study provides the first evidence implicating a causal link between pre-natal exposure to malaria, C5a-C5aR signaling and subsequent neurocognitive impairment in offspring. Our findings indicate that *in utero* exposure to maternal malaria infection can alter the cognitive and neurological development of offspring. We observed impaired learning and memory and depressive-like behavior that persisted to adulthood in EMIP-exposed offspring that were neither congenitally infected nor LBW. These neurocognitive impairments were associated with decreased tissue levels of major biogenic amines in cortical and subcortical regions of the brain. Genetic or functional disruption of maternal C5aR signaling restored the levels of BDNF and cerebral biogenic amines and rescued the associated cognitive phenotype observed in EMIP-exposed offspring.

Immunological stress at the maternal-fetal interface is associated with an increased risk of neurodevelopmental disorders in offspring [25,42–44]. MIP is characterized by the accumulation of parasitized erythrocytes and monocytes/macrophages in the intervillous space, creating a localized immune response in the placenta [6]. It is well established that components of the innate immune system, including complement factors, play diverse roles in angiogenesis, inflammation, neurogenesis and neurodevelopment [45,46]. Increased peripheral and placental levels of C5a are observed in women with MIP and are associated with adverse pregnancy outcomes [5,11]. C5a is a potent initiator of pro-inflammatory as well as anti-angiogenic pathways [9,10]. Together these observations are consistent with several potential mechanisms of impaired neurodevelopment including enhanced neuro-inflammation, altered neurovascular development, or dysregulation of complement-mediated neurodevelopmental processes [47].

Complement components are synthesized in the CNS by microglia, astrocytes and neurons and may be overexpressed in response to injury or inflammation [46,48]. Neurons, unlike peripheral cell types, do not express high levels of complement regulatory proteins, such as CD59, CD46, CD55 and CD35, suggesting that they may be particularly susceptible to complement-mediated injury [49]. A growing body of evidence supports an important role for the complement system in normal neurodevelopment, synapse formation and synaptic pruning [20,21,23]. Complement components tag excess synapses for elimination during pruning, facilitating the formation of mature patterns of neuronal connectivity [20]. Reduced levels of complement have been associated with decreased levels of synaptic pruning in the hippocampus and neocortex, a process critical for synaptic refinement during development [20,21,50]. These findings suggest that increased complement activation during development, as occurs in MIP, could lead to excessive synapse elimination and altered neuronal connectivity [23]. Moreover in models of lipopolysaccharide (LPS)-induced preterm birth C5a is reported to have direct neurotoxic effects on fetal cortical neurons *in vivo* and *in vitro* [22]. These neurotoxic effects were associated with C5a-induced glutamatergic excitotoxicity [22]. Collectively these data support the hypothesis that MIP-induced complement activation at the maternal-fetal interface may alter fetal neural networks and disrupt normal brain developmental processes. While increased peripheral and placental levels of C5a are observed in women with MIP [5,11], it is unclear whether fetal complement activation also occurs and whether it contributes to altered neurodevelopment. Future studies using the EMIP model could examine this question by mating heterozygote parents to generate WT, heterozygote and *C5ar-/-* offspring and determining the relative contribution of maternal versus fetal complement activation to neurocognitive outcome.

We propose that the cognitive deficits we observed in EMIP-exposed offspring are mediated, at least in part, by a reduction in regional brain levels of biogenic amines. Biogenic amines are reported to be central to learning and memory in the NOR test and depressive-like behavior in the TST [30,37,51]. We observed reduced serotonin and dopamine in the frontal cortex, reduced norepinephrine in the temporoparietal cortex and reduced serotonin in the striatum of EMIP-exposed WT offspring. EMIP-exposed offspring do not develop reductions of biogenic amines, in these regions, when C5aR signaling is disrupted. Norepinephrine has been associated with arousal and attention; responses to novelty that facilitate object recognition [33,51]. Cortical monoamine function, including dopamine and serotonin, has also been linked to performance in the NOR test [36,38,52,53]. Previous studies have implicated altered basal ganglia and cortical monoamine levels in TST behavior [37,54]. Specifically, pharmacological treatment with monoamine reuptake inhibitors that increase monoamine availability induce increased mobility in the TST [30,54,55]. Based on our behavioral and HPLC data we postulate that *in utero* exposure to malaria induces localized and subtle changes in neuronal development. We did not observe any behavioral deficits in the CFC test, a test of learning and memory using a highly salient and aversive foot-shock stimulus [56]. This suggests that prenatal exposure to EMIP does not induce a global impairment in learning and memory but alters behavioral performance in a task-specific manner.

Tight regulation of neurotrophic factors, in particular BDNF, is critical for normal neurodevelopment [40,57]. During embryogenesis BDNF regulates axonal and dendritic differentiation [40]. Immune responses to infections may alter BDNF levels, and disruption in BDNF-regulated processes can lead to alterations in brain monoamine levels and behavioral phenotypes in adulthood [41,58]. Our data, together with the above observations, support a model of pathogenesis whereby MIP-induced C5 activation impairs *in utero* neurodevelopment via effects on inflammation, synaptic pruning, neural network formation and regulation of BDNF, leading to reduced regional levels of monoamines and impaired cognitive performance in malaria-exposed offspring. When C5a-C5aR signaling is disrupted by genetic or functional approaches, there is reduced neurotoxicity, preserved regulation of BDNF and brain monoamine levels, and improved neurocognitive outcomes.

In addition to a role in neurodevelopment and neurodegenerative disorders, C5a is a potent initiator and amplifier of anti-angiogenic pathways and could theoretically alter neurodevelopment through angiogenic pathways as has been proposed for placental vascular development and remodeling during MIP [5,10,11]. Since C5a-C5aR blockade has been show to improve placental vascular development, it is also possible that rescue of the cognitive phenotype we observed in malaria-exposed *C5ar-/-* offspring is the result, at least partly, of changes in placental function.

Developmentally, neurogenesis and angiogenesis are tightly linked [59]. They utilize the same genetic and regulatory pathways and dysregulation in one system may alter developmental processes in the other. Therefore, we used a novel imaging approach to investigate whether the neurocognitive deficits in exposed-offspring were linked to altered neurovascular development as proposed [47]. Using micro-CT imaging, we observed an increased number of vessel segments, indicative of more vessel branching in malaria-exposed wild-type offspring. Whether this increase in cerebral vascular development represents a compensatory response to malaria-associated neurotoxicity will require further study. Overall this finding is consistent with previous observations that malaria also alters placental vascular development [5,10,60,61]. However, in the current study, disruption of C5a-C5aR signaling did not significantly reverse the vascular changes and did not provide a clear explanation for the cognitive phenotype observed and its rescue with C5aR blockade

Inflammatory conditions during pregnancy are associated with poor neurodevelopmental outcomes [25,44,62,63]. For example, maternal IL-6 cytokine surges have been reported to induce an increase in the forebrain neural precursor pool via activation of the embryonic neural stem cell self-renewal pathway [64]. Such inflammation-induced changes in early neurogenesis could have a significant impact on cognitive development. We have previously shown that C5a can enhance pro-inflammatory cytokine responses, including IL-6, to malaria-infected erythrocytes [11]. Our data do not exclude a role for neuro-inflammation in EMIP-associated adverse neurocognitive outcomes but rather suggest that both enhanced inflammation and altered neurodevelopment may be mediated through a shared pathway, C5a-C5aR signaling.

In summary, we show that *in utero* exposure to malaria infection disrupts normal cognitive and neurological development of offspring in a model of MIP and implicate activation of C5 in the pathobiology of this phenotype. In the clinical setting, MIP is commonly associated with LBW and there is a well-established link between LBW and increased risk of developmental delay [26,27]. Therefore, the cognitive deficits we observed would be expected to be incrementally increased by other MIP-associated birth complications including LBW caused by fetal growth restriction and preterm birth. Collectively our observations suggest a broader potential impact of malaria exposure *in utero* on neurocognitive outcomes since many malaria infections in pregnancy do not result in an obvious birth phenotype.

Factors that prevent normal neurological development of successive generations of children place enormous financial and social burdens on low resource countries. Persistent neurocognitive impairments as a result of MIP could have broad implications as pregnancies that occur in malaria endemic regions are at risk of MIP [65]. It is essential to identify preventable risk factors that contribute to developmental delay in children. Our data suggest that MIP is one such factor that can be targeted in order to improve cognitive development and school performance in malaria-endemic regions. A prospective study is underway to confirm these findings in children exposed to malaria *in utero* in sub-Saharan Africa (NCT01669941).

## Methods

### Experimental Malaria in Pregnancy (EMIP) model

The EMIP model used in this study was based on a previously validated murine model of MIP, which replicates key pathogenic factors of MIP [28]. Female BALB/c mice (wild-type or *C5ar-/-*) between 6–8 weeks of age were mated with male BALB/c (wild-type or *C5ar-/-*) mice (8–9 weeks) were obtained from Jackson Laboratories (Bar Harbor, ME). *C5ar-/-* females were mated with *C5ar-/-* males, therefore all offspring were also *C5ar-/-*. Naturally mated pregnant mice were infected on G13 with 10^5^ P. *berghei* ANKA-infected erythrocytes in RPMI media via injection into the lateral tail vein. A lower dose of innoculum (10^5^ P. *berghei* ANKA-infected erythrocytes compared with 10^6^) was used in this study to eliminate a low birth weight phenotype and increase the number of live births. Control pregnant females were injected on G13 with RPMI media alone. Thin blood smears were taken daily and stained with Giemsa stain (Protocol Hema3 Stain Set, Sigma, Oakville, ON) to monitor parasitemia. For pharmacological blockade experiments polyclonal rabbit antiserum raised against rat C5a or pre-immune control rabbit antiserum (Sigma G9023) was administered via tail vein injection 2 hours prior to malaria infection (0.25mL) and 72 hours post infection (G16) (0.25mL). Immediately following delivery all pups were given to surrogate (BALB/c wild-type) dams. All mice were weighed weekly beginning at one week of age. All litters were weaned at 3 weeks of age.

### Ethics statement

All experimental protocols were approved by the University Health Network Animal Care Committee (Animal Use Protocol number 1615 5/01/2014) and performed in accordance with the Canadian Council of Animal Care guidelines and current University Health Network regulations.

### Tissue preparation and histology

Placental tissue was collected from uninfected and malaria-infected females at gestational day 19 and whole placentas were immediately fixed in 20x volume of 10% formalin for 48 hours then transferred to 70% alcohol. Paraffin-embedded non-consecutive sections were stained for hemotoxylin-eosin (H&E) and examined under a light microscope (Olympus, BX41, Olympus Corporation).

### Behavioral testing

Behavioral testing was conducted with male offspring beginning at 4 weeks of age and terminating at 7 weeks of age in the order the tests are presented below. In some experiments, testing was performed at 20 weeks. During testing, the experimenter alternated between testing mice from each experimental group. Offspring from a minimum of 4 different litters were used in each testing cohort. All testing was done with the experimenter blinded to the testing group.

#### The Novel Object Recognition test (NOR)

The NOR test is a test of non-spatial, episodic memory that is independent of neuromotor deficits and emotional cues [66]. The test is based on the spontaneous tendency of rodents to explore a novel object over a familiar one. The testing protocol was performed as previously describe [29]. Briefly, mice were habituated to the testing arena (empty clear plastic mouse cages) for 10 minutes over 6 daily sessions. On the test day, each animal was exposed for 10 minutes to a LEGO construct (LEGO Group, Billund, Denmark) and a Hot Wheels car (Mattel, Inc., El Segundo, CA, USA). The objects were previously determined to be of matched saliency for mice. All tests were video recorded suing ANYMAZE software. Time spent exploring both objects was recoded. Exploration was scored when the mouse touched an object with its forepaws or snout, bit, licked or sniffed the objects from a distance of no more than 1.5cm. Following exploration mice were returned to their home cage. Three hours after the initial exposure, mice were returned to the test cage and were exposed for 5 minutes to one object from the original test pair and to a novel object. Any difference between the right and left side of the cage was addressed by counterbalancing for placements of the new object between mice in a test group in both tests. A “preference index” (PI) will be calculated as: PI = tn/(tn + tf), wherein “tn” represents time exploring a novel object or object in a novel placement area and “tf” the duration of familiar object exploration [29]. All animals that explored objects for less than 10 seconds were removed from analysis.

#### The Tail Suspension Test (TST)

The TST is a well-validated murine model of affective behavior [31]. Animals with higher levels of depressive-like behaviors show increased immobile (freezing) behaviour compared to animals with normal baseline levels of depressive-like behaviour that show more movement during testing. The testing protocol was performed as previously described [30,66]. Each mouse was suspended by a small piece of masking tape on the tail for a 6-minute duration. All tests were video recorded using ANYMAZE software. The freezing (immobility) and mobility were coded during testing.

#### The Contextual Fear Conditioning (CFC) test

The CFC test is used to assess learning and memory dependent upon hippocampal (spatial learning) and amydala (emotional, cued learning) function [56]. All testing was conducted using a computer-controlled fear conditioning system (TSE, Bad Homburg, Germany). Fear conditioning took place in a plexiglass chamber (20 cm x 20 cm x 36 cm) within a fear-conditioning box that was under constant illumination. During the testing, mice were single-housed and were brought into the testing room individually. The conditioning trial (Day 1) consisted of a single trial in which the mouse was placed in the test chamber (conditioning context) for 180 sec, after which a 30 sec tone was played (10 kHz, 75 dB SPL). Termination of the tone coincided with the onset of a 2 sec shock (0.7 mA, constant current) delivered through a stainless steel grid floor. The mouse was left in the chamber for a further 30 sec, so that handling upon removal from the testing chamber would not be associated with shock. Contextual memory was assessed 24 h after the conditioning trial (Day 2). Mice were returned to the chamber and left for 210 sec. Conditioned memory was assessed 48 h after completion of the conditioning trial (Day 3). Mice were again returned to the testing chamber. The context was altered by covering the stainless steel rods on the floor with smooth plastic and covering the chamber walls with paper towel. After 180 sec in the chamber, the tone was played for 180 sec. Across days 1–3, total freezing (as measured by total number of light beam breaks) was recorded by the fear conditioning system.

### In vitro magnetic resonance imaging

A separate group of male offspring were behaviourally tested in the NOR test and TST at 5–6 weeks (Fig 1) and were euthanized at 8 weeks of age for MRI. All animals were weighed prior to behavioral testing and prior to perfusion. To minimize the likelihood of neurological changes resulting from behavioral tests using aversive stimuli, as in the CFC test, offspring to be perfused for MRI were only tested in the NOR and TST.

#### Perfusion procedure

At 8 weeks of age animals were anesthetized with a ketamine (150mg/kg)/xylazine (10mg/kg) mix and perfused transcardially with 30 mL of solution A (1xPBS + 2mM ProHance + 1μL/mL Heparin) and then with 30 mL of solution B (1xPBS + 4% Paraformaldehyde + 2mM ProhHance). Animals were then decapitated and skin, cartilage and lower jaw was removed. Tissue was left at 4°C for 24 hours in 10 mL of solution B and then transferred into 10 mL of solution C (1xPBS + 0.02% sodium azide + 2mM ProHance) for storage prior to scanning. Tissue was left in solution C for a maximum of 6 months prior to scanning.

#### MRI acquisition

The MRI methods used here have previously been described in detail [67]. Briefly, a multi-channel 7.0-T MRI scanner (Varian Inc., Palo Alto, CA) containing a 40-cm diameter bore magnet scanned perfused brain tissue within the skulls. Prior to imaging samples were removed from solution C, blotted and placed into 13mm (diameter) plastic tubing filled with a proton-free susceptibility-matching solution (Florinert FC-77, 3 M Corp., St. Paul, MN). Custom-built, solenoid coils were used to image multiple specimens at one time. Scan parameters were set to optimize grey/white matter contrast: T2 weighted, 3D fast spin echo sequence with TR = 2000 ms, echo train length = 6, TE_eff_ = 42 ms, field-of-view (FOV) = 25 x 28 x 14 mm and matrix size = 450 x 504 x 250, resulting in a final image with 56 μm isotropic voxel. Total scan time was 11.7 hours.

#### Image analysis

Four specimens were removed from analysis due to substantial tissue damage sustained during initial tissue collection. Imagine analysis was performed as previously described [68]. Briefly a group wise image registration approach was used where a smooth spatial transformation aligns images to one another so that corresponding anatomical features are superimposed. An average image of all scanned brains is then generated. Local deformation of all brains brings each scan into exact correspondence with the group “average” brain. The deformation required to bring scans into alignment with the average brain is needed to compute the volumetric difference between each specimen image and the average brain. Greedy symmetric diffeomorphic registration (the SyN algorithm in ANTS) was used to calculate the final deformation fields. The fields were then inverted and blurred with a 100-μm Gaussian smoothing kernel and the Jacobian determinants of the deformations were generated, resulting in a measure of local expansion/contraction. A false discovery rate of 10% was used to control for multiple comparisons across the different brain regions. An anatomical mouse brain atlas [32] was used to define brain regions and structures and to compute volumes for each mouse brain.

### Microwave fixation and high-pressure liquid chromatography procedures

Microwave fixation of tissue was used to examine biochemical changes in biogenic amines as they relate to treatment group and performance in behavioral paradigms. All mice were tested prior to microwave fixation in the NOR test and TST. The microwave fixation procedures used here have been previously described in detail [30]. Briefly, all mice were euthanized at 8 weeks of age with a brief pulse (~0.9s) of high intensity microwave radiation (8 kW, 60Hz, 56 Amp) focused to the head and administered by a 10 KW magnetron (model TMW-4012C, Muromachi Kikai, Tokyo, Japan). Microwave fixation allows for rapid heat-inactivation of enzymes *in situ* and avoids confounding results due to *post-mortem* changes. Immediately following heat-inactivation, the heat-inactivated brains were dissected regionally on ice and stored at -80°C prior to analysis. Levels of dopamine (DA), norepinephrine (NE), serotonin (5-HT) and metabolites were assayed in perchloric acid tissue extracts with a Dionex HPLC system and electrochemical detector (DIONEX, Sunnyvale, CA, USA). Biogenic amines were selected based on extensive evidence linking these neurotransmitters with learning, memory and behavioural performance. HPLC was performed only on tissue from the temporoparietal cortex, frontal cortex, striatum, hippocampus and cerebellum based on the well-established role of these specific regions in learning, memory and motor behavior which impact performance in the NOR and TST [30,36,37,51,54]. As described previously, the chromatographic conditions included a C18 reverse-phase column (Acclaim 120, 150 x 4.0 mm^2^ cartridge, 5 μm particle size) at 30°C. The mobile phase consisted of sodium acetate (100 mM) tetrasodium EDTA (0.125 mM), 1-octane sufonic acid (432 mg/l) and 5.0% methanol (final pH = 3.6), delivered at a flow rate of 0.75 mL/min with a UltiMate 3000 pump. Samples (25 μl) were injected automatically with a refrigerated autosampler (UltiMate 3000 autosampler). The electrochemical detection (ESA Coulochem III 5011A analytical cell with a 5020 guard cell) was conducted at a working electrode potential of -400 mV.

### Fetal brain vasculature analysis

Uteri were extracted from dams at gestational day 18 and anesthetized via hypothermia (immersion in ice-cold PBS). Each individual fetus is then extracted from the uterus while maintaining the vascular connection to the placenta. The embryo is briefly resuscitated via immersion in warm PBS to resume blood circulation. Embryo’s that could not be resuscitated are not perfused and were removed from the study. A catheter is then inserted into the umbilical artery and the fetus is perfused with saline (with heparin, 100units/mL) followed by radio-opaque silicone rubber contrast agent (Microfil; Flow Technology, Carver, MA). The perfusions were performed using two different lots of Microfil. Following perfusion specimens are post-fixed with 10% Formalin and imaged using micro-computed tomography (micro-CT). Specimens were scanned at 7.6 um resolution for 1 hour using a Bruker SkyScan1172 high resolution Micro-CT scanner. 996 views were acquitted via 180-degree rotation with an X-ray source at 54 kVp and 185 uA. Three-dimensional micro-CT data were reconstructed using SkyScan NRecon software. Each micro-CT image was manually masked to exclude extracerebral vessels using a cerebral vascular atlas as a guide [69]. The structure of the vasculature was identified automatically using a segmentation algorithm as described in detail previously [35]. Images that showed evidence of rupture of a major vessel or incomplete perfusion of Microfil were excluded from the analysis (9 of 38 specimens, S3 Table). Univariate ANCOVAs were conducted to compare the number of vessel segments and the total length of all vessel segments as a function of group, with dataset as a covariate (to control for the variance from using different lots of Microfil). A linear model was used to estimate the effect of dataset and the total segments and length were adjusted accordingly. Tukey contrasts were used to test differences between the adjusted means. Analysis was performed on wild-type (unexposed (n = 8) and malaria exposed (n = 7)) and C5aR knock out mice (unexposed (n = 7) and malaria exposed (n = 7)).

### Fetal brain and placental tissue transcript analysis

RNA extraction was performed on snap-frozen fetal brain tissue and placental tissue collected at G19. The EMIP model followed the same protocol outlined above. Dams were sacrificed at G19, yolk sacs were dissected from uteri, fetuses were removed and weighed, and fetal brain tissue and placentas were snap frozen and stored at -80°C until analyzed. Fetal viability was determined by assessing pedal withdrawal reflex. Non-viable fetuses (i.e., lacking the pedal withdrawal reflex) were considered aborted. Only viable fetuses and placentas from viable fetuses were used in the analysis. Tissue was homogenized in TRIzol (0.5mL/100mg tissue; Invitrogen, Burlington, ON) according to the manufacturer’s protocol and RNA was extracted. Extracted RNA (2 μg per sample) was then treated with DNase I (Ambion, Streetsville, ON) and reverse transcribed to cDNA with SuperScript III (Invitrogen, Burlington, ON) in the presence of oligo (dT) primers (Fermentas, Burlington, ON) with sequences listed below. Residual RNA was degraded with RNase H (Invitrogen, Burlington, ON). Sample cDNA was amplified in triplicate with SYBR Green master mix (Roche, Laval, QC) in the presence of 1 μM both forward and reverse primers in a Light Cycler 480 (Roche, Laval, QC). Transcript number was calculated based on Ct compared to the standard curve of mouse genomic DNA included on each plate by Light Cycler 480 software (Roche, Laval, QC), and expression in fetal brain was normalized to geometric average of the housekeeping genes GAPDH and β-actin expression levels. Expression in placental tissue was normalized to the housekeeping genes GAPDH and HPRT. A normalization factor was generated for each sample by dividing the mean sample expression by the mean expression of the housekeeping genes. The expression of each target gene was then divided by the normalization factor for that sample to adjust for experimental variation in gene expression [70]. RPTCR Primer Sequences: (5’–3’): GAPDH: TCAACAGCAACTCCCACTCTTCCA–TTGTCATTGAGAGCAATGCCAGCC, β-actin: GCGCCCATGAAAGAAGTAAAA–TTCGATGACGTGCTCAAAAG, HPRT: GGAGTCTGTTGATGTTGCCAGTA–GGGACGCAGCAACTGACATTTCTA, BDNF: GCGCCCATGAAAGAAGTAAA–TTCGATGACGTGCTCAAAAG. ICAM-1: CGGAAGGGAGCCAAGTAACTG–CGACGCCGCTCAGAAGAA, TNF: GACAGACATGTTTTCTGTCAAACG–AAAAGAGGAGGCAACAAGGTAGAG, IFNγ: TTCTGTCTCCTCAACTATTTCTCTTTG—CCCCACCCCCAGATACAAC, MCP: ACCACAGTCCATGCCATCAC—TTGAGGTGGTTGTGAAAAG

### Statistical analyses

Student’s t-test, one-way ANOVA or ANCOVA (non-parametric Kruskal-Wallis, *P* < 0.05) was used to examine statistical significance between experimental groups where indicated. Post-tests on all groups were conducted using Dunn’s multiple comparison test or Tukey contrasts where indicated (*P* < 0.05).

## Supporting Information

S1 Fig H&E stained placentas from wild-type and *C5ar-/-* females. Placental slices from uninfected wild-type dams **(a)** and from Plasmodium berghei-infected wild-type dams **(b,c)**. Placental slices from uninfected *C5ar-/-* **(d)** and from Plasmodium berghei-infected *C5ar-/-* dams **(e,f)**. Arrowheads show infected erythrocytes. Scale bars represent 20 μm.
(EPS)

S2 Fig mRNA expression of inflammatory factors in placental tissue. Extrapolated cDNA concentration of **(a)** intracellular adhesion molecule-1 (ICAM-1), **(b)** tumor necrosis factor (TNF), **(c)** interferon gamma (IFN-γ) and **(d)** monocyte chemotactic protein (MCP) in placentas from unexposed (UE) and malaria-exposed (EX) wild-type and *C5ar-/-* dams (n = 12 placentas from a minimum of 4 litters/group). Box plots show the median, 95% confidence interval with whiskers denoting the maximum and minimum values. *P < 0.05, **P < 0.01, ***P < 0.005; one-way ANOVA and post-test.
(EPS)

S3 Fig (a) Distance travelled during exploration phase of NOR testing in malaria unexposed (UE, n = 15) and malaria exposed (EX, n = 13) offspring tested at 6 weeks of age. (b) Distance travelled during exploration phase of NOR testing in mice tested at 20 weeks of age, and (c) weight of offspring from one to twenty weeks of age of malaria unexposed (UE, n = 11) and malaria exposed (EX, n = 12) offspring. Performance of a new cohort of offspring used for MRI analysis in the NOR test; (d) preference ratio, (e) total exploration time during exploration phase, (f) distance travelled during the testing phase of NOR testing in malaria unexposed (n = 12) and malaria exposed (n = 12) offspring, and (g) weight of offspring used for MRI imaging from one to 6 weeks of age. ***P < 0.005; T-test Error bars represent means +/- SD.
(EPS)

S4 Fig (a) Distance travelled during exploration phase of NOR testing in wild-type unexposed (WT UE, n = 14) malaria exposed (WT EX, n = 13) wild-type offspring and unexposed (*C5aR-/-* UE, n = 13) and malaria exposed (*C5aR-/-* EX, n = 14) C5a receptor knock out offspring. (b) Maternal parasitaemia (day one to seven post infection) expressed as percent of infected red blood cells (iRBCs) per total red blood cells counted in wild-type (n = 5) and *C5ar-/-* dams (n = 5) with offspring tested at 20 weeks of age. (c) Weight from one to twenty weeks of age in wild-type (WT) malaria unexposed (n = 11), WT malaria exposed (n = 12) *C5ar-/-* unexposed (n = 13) and *C5ar-/-* malaria exposed (n = 9) offspring (d) Distance travelled during exploration phase of NOR testing in the cohort of offspring tested at 20 weeks of age of unexposed (WT UE, n = 11) and malaria exposed (WT EX, n = 12) wild-type offspring and unexposed (*C5aR-/-* UE, n = 13) and malaria exposed (*C5aR-/-* EX, n = 9) C5a receptor knock out offspring. Error bars represent means +/- SD.
(EPS)

S5 Fig (a) Maternal parasitaemia (day one to seven post infection) expressed as percent of infected red blood cells (iRBCs) per total red blood cells counted in wild-type rabbit antisera malaria exposed (n = 4) and C5a antisera treated malaria exposed (n = 4) litters tested in the antibody blockade experiment. (b) Weight from one to six weeks of age in wild-type unexposed (n = 11), malaria exposed offspring (n = 11), malaria exposed offspring of rabbit antisera treated dams (R-Malaria Exposed, n = 12) and malaria exposed offspring of C5a antisera treated dams (αC5a-Malaria Exposed, n = 12). (c) Distance travelled during exploration phase of testing of the NOR test of unexposed offspring (UE, n = 11), malaria exposed offspring (EX, n = 11) malaria exposed offspring of rabbit antisera treated dams (R-EX, n = 12) and malaria exposed offspring of C5a antisera treated dams (αC5a-EX, n = 12). Freezing behaviour in the contextual fear-conditioning test on (d) day 2 (context only) (e) day 3 (conditioning stimulus presentation) pre-tone, and (f) day 3 post-tone of UE (n = 11), EX (n = 10), R-EX (n = 10) and a C5a EX (n = 12) offspring. Error bars represent means +/- SD.
(EPS)

S1 Table Dam peripheral parasitaemia, gestation and litter size from all cohorts. Values represent means ± SD (n = 9–15 per group across all groups).
(PDF)

S2 Table Regional Volumes * by MRI. All volumes in mm^3^ **.**
(PDF)

S3 Table Gross Anatomy Checklist of Fetal Cerebral Vasculature by micro-CT. †Sample was excluded from analysis. *One side of structure is missing due to rupture occurring during perfusion. **Part of the vessel is missing due to incomplete perfusion of Microfil.
(PDF)

S4 Table Regional neurotransmitter content (ng/mg) determined by HPLC in wild type offspring. Values are means +/- SEM (n = 12–15 per group) of the neurotransmitters dopamine (DA), norepinephrine (NE), serotonin (5HT) and the neurotransmitter metabolite homovanillic acid (HVA). Bolded means differ significantly between groups based on a t-test (* p < 0.05. ** p < 0.01).
(PDF)

S5 Table Regional neurotransmitter content (ng/mg) determined by HPLC in *C5ar-/-* offspring. Values are means +/- SEM (n = 13–15 per group) of the neurotransmitters dopamine (DA), norepinephrine (NE), serotonin (5HT) and the neurotransmitter metabolite homovanillic acid (HVA). Bolded means differ significantly between groups based on a t-test (* p < 0.05).
(PDF)
