# Supplementary material for: Experimental Malaria in Pregnancy Induces Neurocognitive Injury in Uninfected Offspring via a C5a-C5a Receptor Dependent Pathway
Source: PLoS Pathog. 2015 Sep 24;11(9):e1005140. doi: 10.1371/journal.ppat.1005140 (PMC4581732; doi:10.1371/journal.ppat.1005140)
Supplement: S1 Table — Values represent means ± SD (n = 9–15 per group across all groups). (PDF) [file ppat.1005140.s006.pdf]

S1 Table: Dam peripheral parasitaemia, gestation and litter size from all cohorts.

		**Unexposed Litters**			**Malaria Exposed Litters**		
**Cohort Presented in Figure:**	**Genotype/ Treatment Group**	**Peripheral Parasitaemia at G19 (%)**	**Gestation (Days)**	**Litter Size (Viable Offspring)**	**Peripheral Parasitaemia at G19 (%)**	**Gestation (Days)**	**Litter Size (Viable Offspring)**
**Figure 1**	WT	0	20.36 ± 0.39	7.25 ± 2.01	17.52 ± 3.03	19.83 ± 0.28	5.83 ± 2.52
	WT (Tested at 20 weeks)	0	19.73 ± 0.45	6.67 ± 2.78	16.24 ± 2.11	20 ± 0	5.90 ± 1.64
**Figure 3**	WT	---------	--------	7.50 ± 1.91	(G18) 25.08 ±5.433	-------	6.58 ±1.27
	C5ar-/-	--------	--------	7.50 ±3.39	(G18) 25.40 ±5.645	-------	7.00 ± 3.46
**Figure 5**	WT	0	19.75 ± 0.25	6.91 ± 2.76	31.12 ± 7.02	20 ± 0	6.08 ± 2.15
	C5ar-/-	0	19.69 ± 0.48	6.00 ± 1.35	14.42 ± 3.35	20.15 ± 0.24	5.00 ± 2.50
	WT (Tested at 20 weeks)	0	19.82 ± 0.22	7.00 ± 1.55	28.86 ± 10.16	19.82 ± 0.36	5.71 ± 2.00
	C5ar-/- (Tested at 20 weeks)	0	20.50 ± 0.46	5.71 ± 0.65	35.19± 12.01	20 ± 0	7.05 ± 2.00
	WT	--------	19.75 ± 0.70	6.91 ± 1.51	31.12 ±8.93	20 ± 0	6.08 ± 1.44
	WT Rabbit Antiserum	--------	-------	--------	8.57 ± 4.47	20 ± 0	6.20 ± 2.10
	WT C5a Antiserum	--------	-------	--------	6.349 ± 3.37	19.92 ± 0.29	8.00 ± 1.35

Values represent means ± SD (n = 9-15 per group across all groups).
