# Supplementary material for: Experimental Malaria in Pregnancy Induces Neurocognitive Injury in Uninfected Offspring via a C5a-C5a Receptor Dependent Pathway
Source: PLoS Pathog. 2015 Sep 24;11(9):e1005140. doi: 10.1371/journal.ppat.1005140 (PMC4581732; doi:10.1371/journal.ppat.1005140)
Supplement: S2 Table — All volumes in mm3 . (PDF) [file ppat.1005140.s007.pdf]

S2 Table: Regional Volumes[*] by MRI

	WT UE		WT EX		*C5aR$^{-/-}$* UE		*C5aR$^{-/-}$* EX	
**Region**	**Mean**	**SD**	**Mean**	**SD**	**Mean**	**SD**	**Mean**	**SD**
Amygdala	12.949	0.906	12.391	0.812	13.698	0.789	13.628	0.862
Anterior Commissure (Anterior)	1.281	0.116	1.258	0.969	1.352	0.074	1.295	0.068
Anterior Commissure (Posterior)	0.521	0.0517	0.483	0.037	0.497	0.062	0.508	0.561
Arbor Vita of Cerebellum	11.499	0.871	11.264	0.639	11.580	0.729	11.585	0.663
Basal Forebrain	5.364	0.400	5.232	0.427	5.370	0.432	5.413	0.442
Bed Nucleus of Stria Terminalis	1.208	0.080	1.147	0.084	1.243	0.089	1.245	0.888
Cerebellar Cortex	49.835	4.138	48.862	3.208	49.731	3.105	48.934	2.783
Inferior Cerebellar Peduncle	0.866	0.040	0.856	0.591	0.869	0.035	0.857	0.059
Middle Cerebellar Peduncle	1.478	0.121	1.454	0.077	1.506	0.064	1.486	0.055
Superior Cerebellar Peduncle	0.899	0.068	0.861	0.049	0.909	0.059	0.927	0.054
Cerebral Aqueduct	0.534	0.074	0.568	0.043	0.555	0.054	0.568	0.077
Cerebral Cortex: Entorhinal Cortex	8.753	0.428	8.490	0.678	8.950	0.572	8.878	0.715
Cerebral Cortex: Frontal Lobe	38.990	2.719	38.328	2.497	41.372	2.374	40.406	2.330
Cerebral Cortex: Occipital Lobe	5.524	0.443	5.314	0.572	5.454	0.496	5.201	0.363
Cerebral Cortex: Parietal Temporal	72.892	4.619	70.642	4.690	75.728	5.106	73.271	3.333
Cerebral Peduncle	2.164	0.143	2.127	0.132	2.267	0.096	2.246	0.117
Inferior Colliculus	5.126	0.382	4.920	0.255	5.234	0.278	5.168	0.510
Superior Colliculus	7.819	0.666	7.623	0.523	7.917	0.524	7.874	0.632
Corpus Callosum	18.160	1.496	17.168	1.331	19.671	1.325	19.244	0.897
Corticospinal Tract (Pyramids)	1.895	0.184	1.813	0.253	1.914	0.160	1.951	0.167
Cuneate Nucleus	0.290	0.042	0.296	0.036	0.292	0.050	0.281	0.052
Hippocampal Dendate Gyrus	3.659	0.255	3.530	0.127	3.837	0.244	3.760	0.191
Facial Cranial Nerve 7	0.245	0.018	0.238	0.015	0.264	0.012	0.256	0.017
Fasciculus Retroflexus	0.247	0.020	0.240	0.019	0.250	0.014	0.246	0.013
Fimbria	2.967	0.252	2.861	0.173	3.131	0.195	3.127	0.207
Fornix	0.631	0.053	0.612	0.038	0.680	0.052	0.660	0.039
Fourth Ventricle	0.422	0.049	0.421	0.034	0.410	0.059	0.400	0.035
Fondus of Striatum	0.180	0.025	0.156	0.011	0.163	0.025	0.173	0.018
Globus Pallidus	2.530	0.151	2.475	0.163	2.712	0.173	2.674	0.148
Habenular Commissure	0.041	0.005	0.044	0.006	0.046	0.003	0.047	0.007
Hippocampus	20.101	1.640	19.581	1.100	21.034	1.233	20.593	1.002
Hypothalamus	8.881	0.578	8.567	0.614	9.047	0.650	9.148	0.631
Inferior Olivary Complex	0.336	0.038	0.310	0.042	0.325	0.029	0.334	0.049
Internal Capsule	2.282	0.210	2.760	0.198	3.020	0.165	2.956	0.150
Interpeduncular Nucleus	0.242	0.022	0.234	0.016	0.229	0.024	0.241	0.019
Lateral Olfactory Tract	1.323	0.086	1.300	0.056	1.395	0.076	1.372	0.137
Lateral Septum	2.418	0.173	2.345	0.175	2.497	0.180	2.512	0.161
Lateral Ventricle	2.279	0.202	2.670	0.240	2.894	0.328	3.014	0.328
Mammillary Bodies	0.576	0.038	0.567	0.050	0.555	0.045	0.569	0.035
Mammilothalamic Tract	0.247	0.018	0.235	0.017	0.253	0.016	0.250	0.013

Medial Lemniscus	2.611	0.179	2.537	0.188	2.629	0.171	2.651	0.205
Medial Septum	1.382	0.098	1.340	0.084	1.394	0.125	1.406	0.126
Medulla	26.696	1.427	26.556	1.395	26.482	1.181	26.487	1.484
Midbrain	12.299	0.854	11.990	0.538	12.641	0.793	12.606	0.797
Nucleus Accumbens	3.706	0.274	3.612	0.263	3.994	0.294	3.949	0.218
Olfactory Bulbs	23.999	2.389	23.171	1.752	25.245	1.883	24.641	2.186
Olfactory Tubercle	3.655	0.399	3.541	0.200	3.460	0.438	3.493	0.444
Optic Tract	1.379	0.112	1.313	0.088	1.463	0.106	1.440	0.753
Periaqueductal Grey	3.886	0.315	3.769	0.226	4.070	0.328	3.977	0.315
Pons	15.406	0.920	15.289	0.886	15.831	0.964	15.761	0.787
Pontine Nucleus	1.163	0.135	1.109	0.094	1.109	0.087	1.121	0.085
Posterior Commissure	0.124	0.015	0.124	0.101	0.138	0.013	0.137	0.014
Preparasubiculum	2.327	0.170	2.276	0.160	2.330	0.111	2.316	0.115
Hippocampus Stratum Granulosum	0.932	0.066	0.889	0.043	0.991	0.083	0.941	0.055
Stria Medullaris	0.697	0.034	0.698	0.037	0.734	0.044	0.723	0.035
Stria Terminalis	0.943	0.066	0.919	0.053	1.000	0.030	0.969	0.047
Striatum	21.678	1.564	21.076	1.433	22.261	1.540	22.045	1.523
Subependymal Zone/Rhinocele	0.0693	0.011	0.065	0.008	0.0800	0.008	0.074	0.010
Superior Olivary Complex	0.667	0.072	0.657	0.063	0.726	0.072	0.718	0.055
Thalamus	15.040	1.163	14.631	0.862	15.590	0.923	15.481	0.848
Third Ventricle	0.872	0.081	0.879	0.088	0.957	0.108	0.965	0.084
Ventral Tegmental Aldecussation	0.115	0.011	0.113	0.007	0.119	0.012	0.122	0.009
Brain Volume	438.320	28.816	426.824	24.844	452.124	24.610	445.347	21.575

*All volumes in mm^3
