# Supplementary material for: Experimental Malaria in Pregnancy Induces Neurocognitive Injury in Uninfected Offspring via a C5a-C5a Receptor Dependent Pathway
Source: PLoS Pathog. 2015 Sep 24;11(9):e1005140. doi: 10.1371/journal.ppat.1005140 (PMC4581732; doi:10.1371/journal.ppat.1005140)
Supplement: S3 Table — †Sample was excluded from analysis. *One side of structure is missing due to rupture occurring during perfusion. **Part of the vessel is missing due to incomplete perfusion of Microfil. (PDF) [file ppat.1005140.s008.pdf]

S3 Table: Gross Anatomy Checklist of Fetal Cerebral Vasculature by micro-CT

Group	ID	Anterior Communicating Artery	Superior Sagittal Sinus	Transverse Sinus	Internal Carotid Arteries	Posterior Cerebral Artery	Basilar Artery	Middle Cerebral Artery
WT UE	3_1_b1416†	Y*	Y	Y	Y	Y	Y**	Y*
	3_4_b1417	Y	Y	Y	Y	Y	Y	Y
	3_5_b1418†	Y	Y	Y	Y*	Y	Y*	Y
	3_6_b1419	Y	Y	Y	Y	Y	Y	Y
	101_1_b1503	Y	Y	Y	Y	Y	Y	Y
	101_3_b1504	Y	Y	Y	Y	Y	Y	Y
	101_5_b1505	Y	Y	Y	Y	Y	Y	Y
	112_3_b1521	Y	Y	Y	Y	Y	Y	Y
	112_4_b1522	Y	Y	Y	Y	Y	Y	Y
	112_5_b1523	Y	Y	Y	Y	Y	Y	Y
WT EX	1_3_b1386†	N	Y	Y	N	N	Y**	N
	1_4_b1387	Y	Y	Y	Y	Y	Y	Y
	7_4_b1388	Y	Y	Y	Y	Y	Y	Y
	1_1_b1385	Y	Y	Y	Y	Y	Y	Y
	104_3_b1500	Y	Y	Y	Y	Y	Y	Y
	104_4_b1501	Y	Y	Y	Y	Y	Y	Y
	104_7_b1502	Y	Y	Y	Y	Y	Y	Y
	111_3_b1513	Y	Y	Y	Y	Y	Y	Y
	111_5_b1514†	Y**	Y	Y	Y	Y**	Y	Y
	111_6_b1515†	Y**	Y	Y	Y**	Y**	Y**	N
C5aR-/- UE	1127_3_b1421	Y	Y	Y	Y	Y	Y	Y
	1127_2_b1420	Y	Y	Y	Y	Y	Y	Y

	1128_8_b1423†	Y**	Y	Y	Y	Y	Y	Y*
	1128_2_b1422	Y	Y	Y	Y	Y	Y	Y
	1376_3_b1506†	Y*	Y	Y	Y	Y	Y	Y*
	1376_4_b1507	Y	Y	Y	Y	Y	Y	Y
	1376_6_b1508	Y	Y	Y	Y	Y	Y	Y
	1390_2_b1518	Y	Y	Y	Y	Y	Y	Y
	1390_3_b1519	Y	Y	Y	Y	Y	Y	Y
	1390_5_b1520†	Y*	Y	Y	Y	Y	Y	Y*
C5aR-/-EX	1080_5_b1395	Y	Y	Y	Y	Y	Y	Y
	1080_6_b1396	Y	Y	Y	Y	Y	Y	Y
	1080_7_b1397	Y	Y	Y	Y	Y	Y	Y
	1081_2_b1393†	Y	Y	Y*	Y	Y	Y*	Y*
	1375_6_b1498	Y	Y	Y	Y	Y	Y	Y
	1375_7_b1499	Y	Y	Y	Y	Y	Y	Y
	1392_1_b1516	Y	Y	Y	Y	Y	Y	Y
	1392_2_b1517	Y	Y	Y	Y	Y	Y	Y

†Sample was excluded from analysis.
*One side of structure is missing due to rupture occurring during perfusion.
**Part of the vessel is missing due to incomplete perfusion of Microfil.
