# Supplementary material for: Experimental Malaria in Pregnancy Induces Neurocognitive Injury in Uninfected Offspring via a C5a-C5a Receptor Dependent Pathway
Source: PLoS Pathog. 2015 Sep 24;11(9):e1005140. doi: 10.1371/journal.ppat.1005140 (PMC4581732; doi:10.1371/journal.ppat.1005140)
Supplement: S4 Table — Values are means +/- SEM (n = 12–15 per group) of the neurotransmitters dopamine (DA), norepinephrine (NE), serotonin (5HT) and the neurotransmitter metabolite homovanillic acid (HVA). Bolded means differ significantly between groups based on a t-test (* p < 0.05. ** p < 0.01). (PDF) [file ppat.1005140.s009.pdf]

S4 Table: Regional neurotransmitter content (ng/mg) determined by HPLC in wild type offspring.

	Unexposed Wild Type Offspring					Malaria Exposed Wild Type Offspring				
	Temporo-parietal Cortex	Frontal Cortex	Striatum	Hippocampus	Cerebellum	Temporo-parietal Cortex	Frontal Cortex	Striatum	Hippocampus	Cerebellum
DA	0.205 ± 0.023	**1.526 ± 0.307** **	5.141 ± 0.347	0.177 ± 0.043	0.033±0.004	0.163 ± 0.272	**1.207 ± 0.307** **	4.473 ± 0.146	0.093 ± 0.009	0.033±0.004
NE	**0.753 ± 0.037** *	0.822 ± 0.039	0.525 ± 0.027	0.703 ± 0.028	0.795±0.024	**0.612 ± 0.042** *	0.822 ± 0.039	0.551 ± 0.036	0.781 ± 0.052	0.767±0.034
5HT	1.077 ± 0.051	**1.183 ± 0.041** **	**1.233 ± 0.022** *	1.478 ± 0.057	0.676±0.049	0.926 ± 0.067	**0.817 ± 0.085** **	**1.095 ± 0.039** *	1.337 ± 0.071	0.565±0.068
HVA	0.078 ± 0.010	**0.454 ± 0.013** **	1.866 ± 0.067	**0.076 ± 0.007** *	0.031±0.002	0.054 ± 0.008	**0.387 ± 0.018** **	1.716 ± 0.064	**0.057 ± 0.005** *	0.036±0.005

Values are means +/- SEM (n = 12-15 per group) of the neurotransmitters dopamine (DA), norepinephrine (NE), serotonin (5HT) and the neurotransmitter metabolite homovanillic acid (HVA). Bolded means differ significantly between groups based on a t-test; *P < 0.05, **P < 0.01.
