# Supplementary material for: Experimental Malaria in Pregnancy Induces Neurocognitive Injury in Uninfected Offspring via a C5a-C5a Receptor Dependent Pathway
Source: PLoS Pathog. 2015 Sep 24;11(9):e1005140. doi: 10.1371/journal.ppat.1005140 (PMC4581732; doi:10.1371/journal.ppat.1005140)
Supplement: S5 Table — Values are means +/- SEM (n = 13–15 per group) of the neurotransmitters dopamine (DA), norepinephrine (NE), serotonin (5HT) and the neurotransmitter metabolite homovanillic acid (HVA). Bolded means differ significantly between groups based on a t-test (* p < 0.05). (PDF) [file ppat.1005140.s010.pdf]

S5 Table: Regional neurotransmitter content (ng/mg) determined by HPLC in *C5ar-/-* offspring.

	Unexposed C5aR-/- Offspring				Malaria Exposed C5aR-/- Offspring			
	Temporo-parietal Cortex	Frontal Cortex	Striatum	Hippocampus	Temporo-parietal Cortex	Frontal Cortex	Striatum	Hippocampus
DA	0.125 ± 0.020	1.325 ± 0.056	4.888 ± 0.421	0.117 ± 0.021	0.160 ± 0.020	1.234 ± 0.212	4.384 ± 0.221	0.110 ± 0.010
NE	0.630 ± 0.037	0.879 ± 0.018	0.595 ± 0.039	**0.872 ± 0.055 ***	0.665 ± 0.042	0.874 ± 0.018	0.620 ± 0.032	**1.002 ± 0.052 ***
5HT	1.116 ± 0.053	1.057 ± 0.039	1.284 ± 0.040	1.372 ± 0.079	1.119 ± 0.065	1.054 ± 0.034	1.302 ± 0.028	1.342 ± 0.085
HVA	0.072 ± 0.015	0.329 ± 0.013	1.569 ± 0.070	0.066 ± 0.016	0.078 ± 0.015	0.331 ± 0.015	1.481 ± 0.054	0.048 ± 0.004

Values are means +/- SEM (n = 13-15 per group) of the neurotransmitters dopamine (DA), norepinephrine (NE), serotonin (5HT) and the neurotransmitter metabolite homovanillic acid (HVA). Bolded means differ significantly between groups based on a t-test (* $p < 0.05$).
